# New records of Sarcophagidae from Turkey (Diptera)

**DOI:** 10.3897/zookeys.703.12377

**Published:** 2017-09-28

**Authors:** Yury Verves, Miroslav Barták, Štěpán Kubík, Hasan Sungur Civelek

**Affiliations:** 1 Institute for Evolutionary Ecology, National Academy of Sciences of Ukraine, Academician Lebedev Str. 37 Kyiv, Ukraine, 03143; 2 Department of Zoology and Fisheries, Faculty of Agrobiology, Food and Natural Resources, Czech University of Life Sciences, Prague, Kamýcká 129, 165 00 Praha Suchdol, Czech Republic; 3 Muğla Sıtkı Koçman University, Faculty of Science, Biology Department, Muğla, Turkey

**Keywords:** distribution, flesh flies, new combinations, new records, new synonyms, Turkey

## Abstract

Faunistic records of 68 flesh fly species are presented, and altogether, 22 species are recorded from Turkey for the first time. A further 46 species were recorded for the first time in at least one Turkish province. This paper presents the first locality data for four additional species, which were previously mentioned only generically in catalogues. One new synonym has been established, Servaisia (s. str.) rybaltschenkoi (Verves, 1977) = *Blaesoxipha ataturkia* Lehrer, 2008, **syn. n.** Two new combinations are proposed: Helicophagella (Parabellieria) dreyfusi (Lehrer, 1994), **comb. n.** and Helicophagella (s. str.) bellae (Lehrer, 2000), **comb. n.**

## Introduction

Sarcophagidae is a large and important family of Calyptrate section of Muscomorpha comprising almost 3,000 species. Adults reach a body length of 2 mm to 22 mm and their larvae have very diverse feeding habits. Some are schizophagous or predacious (i.e., devour other necro- or coprophagous larvae in substrates). The others are obligate or facultative parasitoids of insects (orthopterans, cicadas, beetles, honeybees, etc.), myriapods, terrestrial snails, predators of earthworms, larvae of soil-inhabiting noctuid caterpillars, and pupae of dendrophilous lepidopterans, kleptoparasites of solitary wasps, bees and, to a lesser extent, termites. Certain species are known to prey on the eggs of marine turtles and lizards, as well as spider egg cocoons and oothecae of locusts. Larvae of many species can facultatively produce wounds or occasional intestinal myiasis on various vertebrates, including humans. Obligate parasites of vertebrates include larvae of several species of *Wohlfahrtia* Brauer & Bergenstamm and some American specialized species with amphibian hosts. Certain synanthropic species are considered mechanical vectors of various intestinal diseases, including poliomyelitis, leprosy, tuberculosis, and mycosis (Rohdendorf 1937; Pape 1996; Povolný and Verves 1997; Verves 1986a; Verves and Khrokalo 2006a, b, 2015; Verves et al. 2015a).

The first reviews of Turkish Sarcophagidae were prepared by Verves (1986a, b) and Kara and Pape (2002); they listed a total of 85 and 81 species, respectively. Many authors have, through various studies, since brought the number of known Turkish species to 137 (Civelek and Tezcan 2005; Lehrer 2006; Aslan 2006; Hayat et al. 2008; Aslan and Çalişkan 2009; Gözüaçik and Mart 2009; Karaman et al. 2009; Pekbey and Hayat 2010; Xue et al. 2011, 2015; Koçak 2014; Koçak and Kemal 2009, 2012, 2013, 2015; Whitmore 2011; Pekbey and Hayat 2011, 2013a, b, c; Whitmore et al. 2013; Verves & Khrokalo 2015; Verves et al. 2015b; Kemal and Koçak 2015; Pekbey 2011; Pekbey et al. 2011 a, b). Other studies provided detailed data for the species causing obligate myiasis [*Wohlfahrtia magnifica* (Schiner)], and several facultative myiasis-causing species (Kurtpinar 1950; Dinçer 1997; Çiftçioglu et al. 1997; Şaki and Özer 1999a, b; Dinçer et al. 2001; Sevgili et al. 2004; Kökçam and Şaki 2005; Yuca et al., 2005; Ütük 2006; Büyükkurt et al. 2008; Aydenizöz and Dik 2008; Yildirim et al. 2008; Yazgi et al. 2009; İpek et al. 2009; Tuygun et al. 2009; Bayındır et al. 2010; İpek and Şaki 2010; Akduman et al. 2010; Kara and Arslan 2011; Dik et al. 2012; Övet et al. 2012; Kılınç et al. 2013; Ozsoy et al. 2013; Köse et al. 2013; Çevik et al. 2014; Özdemir et al. 2014; Gümüşsoy et al. 2015). The true number of Turkish sarcophagids may range from 175 to 250 species. The main aim of the present paper is to capitalize on the results of several expeditions to Turkey and in such a way to enrich knowledge on the flesh fly fauna of Turkey.

## Materials and methods

The material examined in this study originates from southwestern Turkey, mainly from the Muğla province but also, to a minor extent, from the four adjacent provinces of Aydın, Burdur, Denizli, and Antalya and from the Samsun province. Most of the material was obtained during field expeditions of M. Barták and Š. Kubík using Malaise traps (**MT**) and yellow pan traps (**PT**), and by sweeping of vegetation (**SW**). Some specimens were collected by the senior author (**YV**) in southern Turkey in 2010–2011. Most of the specimens were originally preserved in alcohol and later dried and mounted using the method described by Barták (1997).

All specimens were identified by the senior author using numerous published keys, descriptions, and illustrations (Rohdendorf 1955, 1971, 1975, 1988; Verves 1982a, 1982b, 1985, 1989a, 1989b, 1993, 1994, Verves and Khrokalo 2006a, Whitmore 2011, Xue et. al. 2011). In this paper, we included only reliably identified species. Specimens with uncertain identity and/or belonging to undescribed species will be published at a later date. We follow the classification scheme of Rohdendorf (1965, 1967) with subsequent additions of Verves (1986), Povolný and Verves (1997), Verves & Khrokalo (2006a, b, 2009, 2015), Verves et al. (2015b), Xue et al. (2011, 2015).

Distributional data of sarcophagids in Turkey were derived from the analysis of all available publications (see reference list). General species distribution was derived from Pape (1996, 2015), Povolný and Verves (1997), Verves & Khrokalo (2006a, b, 2009, 2015), Verves et al. (2015b), Xue et al. (2011, 2015) and other sources (as indicated under several species treated below). Classification of distributional ranges follows Gorodkov (1983, 1984).

Voucher specimens are deposited at **CULSP** (Czech University of Life Sciences, Prague, Czech Republic) and **IEE** (Institute for Evolutionary Ecology, National Academy of Sciences, Kyiv, Ukraine). Other abbreviations are used in text as follows: **DI** istribution, **DIT** distribution in Turkey, **TR** Turkey (species listed in catalogs without specification of locality data). Provinces are abbreviated as follows:

**AD** (Adana), **AF** (Afyonkarahisar), **AM** (Amasya), **AN** (Ankara), **ANT** (Antalya), **AY** (Aydın), **BT** (Batman), **BY** (Bayburt), **BO** (Bolu), **BU** (Burdur), **CA** (Çanakkale), **DE** (Denizli), **DB** (Diyabakir), **DU** (Düzce), **ED** (Edirne), **EL** (Elazığ), **ER** (Erzincan), **ERZ** (Erzurum), **ES** (Eskişehir), **GA** (Gaziantep), **HA** (Hakkari), **HT** (Hatay), **IG** (Iğdır), **IZ** (İzmir), **KM** (Karaman), **KAR** (Kars), **KY** (Kayseri), **KI** (Kırıkkale), **KK** (Kırklareli), **KN** (Konya), **MN** (Manisa), **MR** (Mardin), **ME** (Mersin), **MG** (Muğla), **SA** (Samsun), **SN** (Şanlıurfa), **TO** (Tokat), **TB** (Trabzon), **VA** (Van).

## Results

Species newly recorded from Turkey are marked with an asterisk (*), and from individual provinces with two asterisks (**). Localities are listed alphabetically according to province and locality names.

### Subfamily Miltogramminae

#### Tribe Miltogrammini

##### Subtribe Senotainiina

**Senotainia (Arrenopus) albifrons (Rondani, 1859)**

**Material examined. AY**: 8 km S of Çine, river bank, SW, 68 m, 37°32'34"N, 28°03'46"E, 28.–30.vi.2015 (Barták & Kubík), 3 ♂, 3 ♀; **MG**: Akyaka, pasture, 4 m, 37°03'09"N, 28°20'17"E, 8.–14.ix.2014 (Barták & Kubík), 1 ♂; ibid., salty meadow, 2 m, PT, 37°01'49"N, 28°20'01"E, 22.vi.–1.vii.2015 (Barták & Kubík), 17 ♂, 17 ♀; ibid., 37°01'62"N, 28°20'00"E, 27.iv.–1.v.2016 (Barták & Kubík), 3 ♀; Dalyan, farm, MT, 1 m, 36°48'54"N, 28°39'04"E, 8.–20.viii.2015 (Dursun), 1 ♂, 1 ♀ Muğla Univ. campus, SW+PT, 700 m, 37°09"39"N, 28°22"20"E, xi.–iii.2013 (Barták & Kubík), 1 ♀; ibid., 730 m, MT, 37°09'38"N, 28°22'11"E, 5.–19.viii.2015 (H. Kavak), 1 ♂; **SA**: Samsun University campus, 41°22'N, 36°11'E, 22vi.–4.vii.2014 (Barták & Kubík), 14 ♂, 1♀ (CULSP).

**DI**: Trans-Palaearctic-Afrotropical-Oriental.

**DIT**: **ANT** (Koçak and Kemal 2009, 2012, 2015; Kara and Pape 2002), AY**, MG**, SA**.

##### Subtribe Miltogrammina

*Miltogramma aurifrons* Dufour, 1850*

**Material examined. MG**: Muğla University campus, Malaise trap, 700 m, 37°09'42"N, 28°22'21"E (O.Dursun), v.2013, 1 ♂; ibid., SW+PT, 37°09'42"N, 28°22'21"E, 29.iv.–10.v.2013 (Barták & Kubík), 2 ♂; ibid., 730 m, 37°09'38"N, 28°22'11"E, xi.2015–iv.2016 (Barták & Kubík), 2 ♀; Muğla – 13 km NE, pine wood, 1200 m, 37°14'50"N, 28°30'00"E, 23–27.v.2016 (Barták & Kubík), 1 ♀ (CULSP).

**DI**: Mediterranean.

**DIT**: MG**.

*Miltogramma brevipila* Villeneuve, 1911*

**Material examined. AY**: 8 km S of Çine, river bank, 68 m, 37°32'34"N, 28°03'46"E, 10.–12.ix.2014 (Barták & Kubík), 1 ♀ (CULSP).

**DI**: West-Central Palaearctic.

DIT: AY**.

***Miltogramma murina*** Meigen, 1824

**Material examined. MG**: Muğla University campus, MT, 730 m, 37°09'38"N, 28°22'11"E, xi.2015–iv.2016, (Barták & Kubík), 1 ♀ (CULSP).

**DI**: West Palaearctic.

**DIT**: **TR** (Povolný and Verves 1997), MG**.

*Miltogramma testaceifrons* (Roser, 1840)*

**Material examined. MG**: Muğla University campus, MT, 730 m, 37°09'38"N, 28°22'11"E, xi.2015–iv.2016 (Barták & Kubík), 1 ♀ (CULSP).

**DI**: West Palaearctic-Oriental.

**DIT**: MG**.

*Miltogramma turkmenora* Rohdendorf, 1930*

**Material examined. MG**: Muğla University campus, 700 m, SW+PT, 37°09'42"N, 28°22'21"E, 29.iv.–10.v.2013 (Barták & Kubík), 4 ♂, 4 ♀ (CULSP).

**DI**: Western Middle East.

**DIT**: MG**.

##### Subtribe Apodacrina

*Apodacra dispar* Villeneuve, 1916*

**Material examined. AY**: 8 km S of Çine, river bank, 68 m, 37°32'34"N, 28°03'46"E, 10.–12.ix.2014 (Barták & Kubík), 1 ♂; **MG**: Muğla University campus, 710 m, MT, 37°09'39"N, 28°22'20"E, xi–iii.2013 (Barták & Kubík), 7 ♀; ibid., 730 m, 37°09'38"N, 28°22'11"E, xi.2015–iv.2016 (Barták & Kubík), 6 ♀ (CULSP).

**DI**: West Palaearctic-Afrotropical.

**DIT**: AY**, MG**.

##### Subtribe Craticulinina

*Craticulina barbifera* (Pandellé, 1895)*

**Material examined. MG**: Akyaka, salty meadow, 2 m, 37°01'49"N, 28°20'01"E, 22.vi.–1.vii.2015(Barták & Kubík), 1 ♀; Dalyan, salty meadow, PT, 36°47'49"N, 28°38'55"E, 28.–30.iv.2016 (Barták & Kubík), 1 ♂ 1 ♀ (CULSP).

**DI**: Mediterranean.

DIT: MG**.

#### Tribe Metopiaini

##### Subtribe Metopiaina

***Metopia argyrocephala* (Meigen, 1824)**

**Material examined. AY**: 8 km S of Çine, river bank, SW, 68 m, 37°32'34"N, 28°03'46"E, 28.–30.vi.2015 (Barták & Kubík), 1 ♂; **MG**: Muğla University campus, SW+PT, 700 m, 37°09'41"N, 28°22'21"E, 29.iv.–10.v.2013 (Barták & Kubík), 3 ♂, 1 ♀; ibid., 720 m, MT, 37°09'42"N, 28°22'13"E, 26.v.–26.vi.2015 (H. Kavak), 1 ♂, 1 ♀; ibid., 26–27.vi.2015 (Barták & Kubík), 1 ♀; **SA**: Samsun, University campus, 41°22'N, 36°11'E, 22vi.–4.vii.2014 (Barták & Kubík), 12 ♂, 1♀ (CULSP).

**DI**: Trans Palaearctic-Nearctic-Oriental-Neotropical.

**DIT**: **TR** (Kara and Pape 2002; Koçak 2014; Koçak and Kemal 2009, 2012, 2015), AY**, MG**, SA**.

*Metopia grandii* Venturi, 1953*

**Material examined. MG**: Akyaka, pasture, 4 m, YPWT, 37°03'09"N, 28°20'17"E, 13.–14.ix.2014 (Barták & Kubík), 1 ♂ (CULSP).

**DI**: Palaearctic.

**DIT**: MG**.

##### Subtribe Taxigrammina

***Paragusia elegantula* (Zetterstedt, 1844)**

**Material examined. MG**: Muğla University campus, 720 m, MT, 37°09'42"N, 28°22'13"E, iv.2015 (H. Kavak), 1 ♀ (CULSP).

**DI**: European-Siberian-Mid-Asiatic.

**DIT**: **TR** (Kara and Pape 2002; Koçak 2014; Koçak and Kemal 2009, 2012, 2013, 2015), MG**.

*Paragusia multipunctata* (Rondani, 1859)*

**Material examined. MG**: Muğla University campus, 710 m, MT, 37°09'39"N, 28°22'20"E, xi.–iii.2013 (Barták & Kubík), 1 ♀; ibid., 720 m, 37°09'42"N, 28°22'13"E, iv.2015 (H. Kavak), 1 ♀ (CULSP).

**DI**: West Palaearctic-Afrotropical-Oriental.

**DIT**: MG**.

***Taxigramma heteroneura* (Meigen, 1830)**

**Material examined. AY**: 8 km S of Çine river bank, 68 m, 37°32'34"N, 28°03'46"E, 10.–12.ix.2014 (Barták & Kubík), 1 ♂, 1 ♀; ibid., 28.–30.vi.2015 (Barták & Kubík), 2 ♂; **MG**: Muğla University campus, MT, 730 m, 37°09'38"N, 28°22'11"E, xi.2015–iv.2016 (Barták & Kubík), 3 ♂, 4 ♀ (CULSP).

**DI**: Trans Palaearctic-Nearctic-Oriental.

**DIT**: **TR** (Kara and Pape 2002; Koçak 2014; Koçak and Kemal 2009, 2012, 2013, 2015), AY**, MG**.

##### Subtribe Sphenometopiina

**Sphenometopa (Xantharaba) steini (Schiner, 1862)**

**Material examined. MG**: Muğla University campus, 700 m, PT, 37°09'42"N, 28°22'21"E (O. Dursun), v.2013, 2 ♀ (CULSP).

**DI**: Eastern Mediterranean.

**DIT**: **TR** (Koçak 2014; Koçak and Kemal 2012), **AN** (Koçak and Kemal 2009, 2013, 2015), MG**.

#### Tribe Phyllotelini

##### Subtribe Arabiscina

*Sphecapatodes ornatus* Villeneuve, 1912*

**Material examined. MG**: Toparlar waterfall, 44 m, 36°59'73"N, 28°38'08"E, 30.v.2009 (O. Dursun), 3 ♂, 1 ♀ (CULSP).

**DI**: East Mediterranean-Midasiatic.

**DIT**: MG**.

### Subfamily Paramacronychiinae

#### Tribe Nyctiini

***Nyctia halterata* (Panzer, 1798)**

**Material examined. AY**: 8 km S of Çine, river bank, 68 m, 37°32'34"N, 28°03'46"E, 10.–12.ix.2014 (Barták & Kubík), 1 ♂; ibid., 28.–30.vi.2015 (Barták & Kubík), 1 ♂; ibid., 29.iv.–1.v.2016 (Barták & Kubík), 2 ♂; **MG**: Akyaka, pasture, YPWT, 37°03'09"N, 28°20'17"E, 4 m, 13.–14.ix.2014 (Barták & Kubík), 1 ♂; ibid., 4 m, 37°03'09"N, 28°20'17"E, 8.–14.ix.2014 (Barták & Kubík), 1 ♂; ibid., salty meadow, 2 m, 37°01'49"N, 28°20'01"E, 22.vi.–1.vii.2015 (Barták & Kubík), 1 ♂, 1 ♀ (CULSP).

**DI**: West Palaearctic.

**DIT**: **TR** (Koçak 2014; Koçak and Kemal 2012), **AM** (Kara and Pape 2002; Koçak and Kemal 2009, 2013, 2015), AY**, MG**, **TO** (Kara and Pape 2002; Koçak and Kemal 2009, 2013, 2015).

***Nyctia lugubris* (Macquart, 1843)**

**Material examined. MG**: Kızılyaka, on flowers, 105 m, 37°01'21"N, 28°26'18"E, 27.iv.–4.v.2016 (Barták & Kubík), 1 ♂, 1 ♀; Akyaka, salty meadow, 2 m, 37°01'49"N, 28°20'01"E, 22.vi.–1.vii.2015 (Barták & Kubík), 5 ♂; ibid., PT, 37°01'62"N, 28°20'00"E, 27.iv.–1.v.2016 (Barták & Kubík), 1 ♂; Dalyan, salty meadow, PT, 36°47'49"N, 28°38'55"E, 28.–30.iv.2016 (Barták & Kubík), 2 ♂ (CULSP).

**DI**: Mediterranean.

**DIT**: **TR** (Koçak 2014; Koçak and Kemal 2012), **BU** (Kara and Pape 2002; Koçak and Kemal 2009, 2013, 2015), MG**, **SA** (Kara and Pape 2002; Koçak and Kemal 2009, 2013, 2015).

#### Tribe Paramacronychiini

##### Subtribe Wohlfahrtiina

*Sarcophila canaanita* Lehrer, 2007*

**Material examined. ANT**: Side, 36°47’38"N, 31°22’43"E, 10.–19.viii.2011 (Yu. Verves), 19 ♂♂, 14 ♀♀ (IEE); **AY**: 8 km S of Çine river bank, 68 m, 37°32’34"N, 28°03’46"E, 10.–12.ix.2014 (Barták & Kubík), 1 ♂; ibid., 28.–30.vi.2015 (Barták & Kubík), 1 ♂; **MG**: Muğla University campus, 720 m, MT, 37°09'42"N, 28°22'13"E, iv.2015 (H. Kavak), 1 ♂; Akyaka, salty meadow, 2 m, PT, 37°01'62"N, 28°20'00"E, 27.iv.–1.v.2016 (Barták & Kubík), 1 ♂; 4 km N of Yatağan, flowers, 460 m, 37°22'12"N, 28°09'22"E, 30.vi.2016 (Barták & Kubík), 3 ♂ (CULSP).

**D**I: East Mediterranean.

**DIT**: ANT**, AY**, MG**.

***Sarcophila latifrons* (Fallén, 1817)**

**Material examined. MG**: MuğlaUniv. campus, protein trap, pine wood, 700 m, 37°09'41"N, 28°22'21"E, xi–iii.2013 (Barták & Kubík), 1 ♂; Toparlar, Toparlar waterfall, 44 m, 36°59'73"N, 28°38'08"E, 30.v.2009 (O. Dursun), 1 ♂ (CULSP).

**DI**: West-Central Palaearctic.

**DIT**: **TR** (Aksoy and Bahadıroğlu 2012; Koçak 2014; Koçak and Kemal 2012), **AD** (Kara and Pape 2002; Koçak and Kemal 2009, 2013, 2015), **ADI** (Gözüaçık and Mart 2009), **BT** (Gözüaçık and Mart 2009), **MR** (Gözüaçık and Mart 2009), MG**, **SN** (Gözüaçık and Mart 2009; Kara and Pape 2002; Koçak and Kemal 2009, 2015).

***Sarcophila meridionalis* Rohdendorf & Verves, 1982**

**Material examined. MG**: Akyaka, pasture, 6 m, 37°03'19"N, 28°20'07"E, 28.iv–8.v.2013 (Barták & Kubík), 1 ♂; ibid. 4 m, 37°03'09"N, 28°20'17"E, 8.–14.ix.2014 (Barták & Kubík), 1 ♂; Muğla University campus, Malaise trap, 700 m, 37°09'42"N, 28°22'21"E (O. Dursun), v.2013, 2 ♂ (CULSP).

**DI**: West-Central Palaearctic.

**DIT**: **TR** (Koçak 2014; Koçak and Kemal 2012), **ER** (Koçak and Kemal 2015; Pekbey 2011; Pekbey and Hayat 2013b), **ERZ** (Koçak and Kemal 2013, 2015; Pekbey 2011; Pekbey and Hayat 2010, 2013b), MG**.

### Subfamily Sarcophaginae

#### Tribe Protodexiini

##### Subtribe Protodexiina

*Blaesoxipha confusa* Villeneuve, 1912*

**Material examined. SN**: Birecik, E from Gaziantep, pastures SE from town, 37.00N/38.00E, 24.–25.iv.1997 (Vrabec V.), 1 ♂ (CULSP).

**DI**: West Palaearctic.

**DIT**: SN**.

***Blaesoxipha redempta* (Pandellé, 1896)**

**Material examined. SA**: Samsun University campus, 41°22'N, 36°11'E, 22vi.–4.vii.2014 (Barták & Kubík), 12 ♂, 1 ♀; **MG**: Toparlar, lowland forest, 8 m, 36°59'27"N, 28°38'50"E, 11.ix. 2014 (Barták & Kubík), 1 ♀ (CULSP).

**DI**: Transpalaearctic-Afrotropical-Oriental-Australasian/Oceanian (Hawaii, imported: Hardy, 1980).

**DIT**: **TR** (Kara and Pape 2002; Koçak 2014; Koçak and Kemal 2009 – as *B. lapidosa*), **BY** (Pekbey 2011; Pekbey and Hayat 2013c), **CA** (Calvert 1882), **ER** (Pekbey 2011; Pekbey and Hayat 2013c), **ERZ** (Pekbey 2011– as *B. lapidosa*; Pekbey and Hayat 2010, 2013c), MG**, SA**.

Comment: Analysis of Pape’s (1994) description of *B. lapidosa* and redescriptions of *B redempta* by Leonide and Leonide (1983) and Pape (1994), our opinion is such: *B. lapidosa* is a synonym of *B. redempta*, because the original descriptions and drawings of ♂ genitalia and ovipositors of both species are very detailed and essentially not differentiated from each other.

**Servaisia (s. str.) rybaltschenkoi (Verves, 1977)**

*Blaesoxipha ataturkia* Lehrer, 2008a, **syn. n.**

**DI**: European-Anatolian-Midasiatic.

**DIT**: TR (Koçak 2014 – as *Blaesoxipha ataturkia*; Koçak and Kemal 2012), **HA** (Kemal and Koçak 2015 – as *Blaesoxipha ataturkia*; Koçak and Kemal 2013– as *Blaesoxipha ataturkia*; Lehrer 2008a).

**Taxonomic notes**: The original descriotion and drawings of ♂ genitalia of male *Blaesoxipha ataturkia* are very detailed and essentially not diﬀerentiated from similar descriptions and drawings of ♂ genitalia of *Blaesoxipha rybaltschenkoi* Verves, 1977. The diﬀerences in drawings are very petty and reﬂected the diﬀerent styles of painters; they cannot be used as reason for separation *B. ataturkia* from *S. rybaltschenkoi*.

#### Tribe Johnsoniini

##### Subtribe Sarcotachinellina

***Sarcotachinella sinuata* (Meigen 1826)**

**Material examined. MG**: Akyaka, pasture, 6 m, 37°03'19"N, 28°20'07"E, 28.iv.–8.v.2013, YPWT (Barták & Kubík), 5 ♂; ibid., pasture, YPWT, 37°03'09"N, 28°20'17"E, 4 m, 13.–14.ix.2014 (Barták & Kubík), 1 ♂; ibid., salty meadow, 2 m, PT, 37°01'62"N, 28°20'00"E, 27.iv.–1.v.2016 (Barták & Kubík), 1 ♂ (CULSP).

**DI**: Transpalaearctic-Nearctic [Holarctic].

**DIT**: **TR** (Koçak 2014; Koçak and Kemal 2012), **AM** (Kara and Pape 2002; Koçak and Kemal 2009, 2013, 2015), **ER** (Pekbey 2011), **ERZ** (Pekbey 2011), **KY** (Hayat et al. 2008; Koçak and Kemal 2009, 2013, 2015), MG**, **TO** (Kara and Pape 2002; Koçak and Kemal 2009, 2013, 2015).

#### Tribe Raviniini

##### Subtribe Raviniina

***Ravinia pernix* (Harris, 1780)**

**Material examined. AY**: 8 km S of Çine, river bank, 68 m, 37°32'34"N, 28°03'46"E, 10.–12.ix.2014 (Barták & Kubík), 2 ♂; ibid., 28.–30.vi.2015 (Barták & Kubík), 1 ♂; ibid., 29.iv.–1.v.2016 (Barták & Kubík), 1 ♂; **MG**: Muğla Univ. campus, protein trap, pine wood, 700 m, 37°09'41"N, 28°22'21"E, xi.–iii.2013 (Barták & Kubík), 1 ♂; University campus, SW+PT, 700 m, 37°09'41"N, 28°22'21"E, 29.iv.–10.v.2013 (Barták & Kubík), 1 ♂ (CULSP).

**DI**: Transpalaearctic-Afrotropical-Oriental.

**DIT**: **TR** (Koçak 2014; Koçak and Kemal 2012), **AD** (Aslan and Çalışkan 2009; Kara and Pape 2002;Koçak and Kemal 2009, 2013, 2015), AY**, **ER** (Pekbey 2011), **ERZ** (Pekbey 2011; Pekbey and Hayat 2010), **ES** (Aslan and Çalışkan 2009), **KY** (Hayat et al. 2008; Koçak and Kemal 2009, 2013, 2015), **KN** (Aslan 2006; Hayat et al. 2008; Kara and Pape 2002; Koçak and Kemal 2013, 2015), **ME** (Aslan 2006; Kara and Pape 2002; Koçak and Kemal 2009, 2013, 2015), MG**, **SN** (Sevgili et al. 2004), **TO** (Aslan 2006; Hayat et al. 2008; Kara et Pape 2002; Koçak and Kemal 2009, 2013. 2015).

#### Tribe Sarcophagini

##### Subtribe Helicophagellina

Helicophagella (Parabellieria) macrura (Rohdendorf, 1937)*

**Material examined. AY**: 9 km S of Çine River bank, 70 m, 37°31'36"N, 28°04'29"E, 2.v.2013 (Barták & Kubík), 1 ♂ (CULSP).

**DI**: Transpalaearctic subboreal.

**DIT**: AY**.

**Helicophagella (Parabellieria) melanura (Meigen, 1926)**

**Material examined. ANT**: Alanya, Avsallar, 36°36"54"N, 31°46"38"E, ground path in bushes, 4–7.viii.2010, (Yu. Verves), 1 ♀; Antalya, Side, clay loam waste plot of land, 36°47"38"N, 31°22"43"E, 10–19.viii.2011 (Yu. Verves), 6 ♂♂, 7 ♀♀ (IEE); **AY**: 9 km S of Çine river bank, 70 m, 37°31'36"N, 28°04'29"E, 2.v.2013 (Barták & Kubík), 2 ♂; ibid., 68 m, 37°32'34"N, 28°03'46"E, 10.–12.ix.2014 (Barták & Kubík), 5 ♂; ibid., 28.–30.vi.2015 (Barták & Kubík), 2 ♂; **MG**: Akyaka, pasture, 2 m, 37°03'09"N, 28°20'17"E, YPWT, 23–27.ix.2012 (Barták & Kubík), 1 ♀; ibid., 6 m, YPWT, 37°03'19"N, 28°20'07"E, 28.iv.–8.v.2013 (Barták & Kubík), 3 ♂; ibid., salty meadow, SW+PT, 37°02'53"N, 28°19'39"E, 28.iv.–9.v.2013 (Barták & Kubík), 1 ♂; ibid., pasture, YPWT, 37°03'09"N, 28°20"17'E, 4 m, 13.–14.ix.2014 (Barták & Kubík), 3 ♂; ibid., 4 m, 37°03'09"N, 28°20'17"E, 8.–14.ix.2014 (Barták & Kubík), 3 ♂; Dalyan, salty meadow, PT, 36°47'49"N, 28°38'55"E, 28.–30.iv.2016 (Barták & Kubík), 1 ♂; **SA**: Samsun University campus, 41°22'N, 36°11'E, 22vi.–4.vii.2014 (Barták & Kubík), 3 ♂ (CULSP).

**DI**: Transpalaearctic-Nearctic-Afrotropical-Oriental.

**DIT**: **TR** (Kara and Pape 2002; Koçak 2014; Koçak and Kemal 2012): ANT**, AY**, **BY** (Pekbey 2011), **ER** (Pekbey 2011), **ERZ** (Pekbey 2011; Pekbey and Hayat 2010), **ES** (Aslan 2006; Aslan and Çalışkan 2009; Koçak and Kemal 2009, 2015), **KY** (Hayat et al. 2008; Kara and Pape 2002; Koçak and Kemal 2009, 2015), MG**, SA**, **SN** (Hayat et al. 2008; Koçak and Kemal 2009, 2015), **TO** (Aslan 2006).

**Helicophagella (s. str.) bellae (Lehrer, 2000), comb. n.**

*Boettcheriola bellae* Lehrer, 2000

**Material examined. MG**: Muğla – 13 km NE, pine wood, 1200 m, 37°14'50”N, 28°30'00”E, 23.–27.vi.2015 (Barták & Kubík), 1 ♂ (CULSP).

**DI**: East Mediterranean.

**DIT**: **TR** (Koçak 2014; Koçak and Kemal 2012): **ANT** (Kara and Pape 2002; Koçak and Kemal 2009, 2013, 2015), **BU** (Kara and Pape 2002; Koçak and Kemal 2009, 2013, 2015), **KM** (Kara and Pape 2002; Koçak and Kemal 2009, 2013, 2015), MG**.

**Helicophagella (s. str.) crassimargo (Pandellé, 1896)**

**Material examined. AY**: 8 km S of Çine river bank, 68 m, 37°32'34"N, 28°03'46"E, 10.–12.ix.2014 (Barták & Kubík), 2 ♂; **MG**: Akyaka, pasture, 6 m, 37°03'19"N, 28°20'07"E, 28.iv.–8.v.2013, YPWT (Barták & Kubík), 6 ♂; ibid., YPWT, 37°03'09"N, 28°20'17"E, 4 m, 13.–14.ix.2014 (Barták & Kubík), 7 ♂; Toparlar, 60 m, lowland wood, SW, 36°58'39"N, 28°39'30"E, 5–7.v.2013 (Barták & Kubík), 1 ♂; ibid., SW+PT, 36°58'39"N, 28°39'30"E, 28.–30.iv.2016 (Barták & Kubík), 2 ♂ (CULSP).

**DI**: European-Siberian-Midasiatic.

**DIT**: **TR** (Koçak 2014; Koçak and Kemal 2012), **AM** (Kara and Pape 2002; Koçak and Kemal 2009, 2013, 2015), AY**, **BY** (Pekbey 2011), **ER** (Pekbey 2011), **ERZ** (Pekbey 2011; Pekbey and Hayat 2010), **KY** (Hayat et al. 2008; Koçak and Kemal 2009, 2013, 2015), MG**, **TO** (Kara and Pape 2002; Koçak and Kemal 2009, 2013, 2015).

Helicophagella (s. str.) novella (Baranov, 1929)*

**Material examined. MG**: Akyaka, pasture, 4 m, 37°03'09"N, 28°20'17"E, 8.–14.ix.2014 (Barták & Kubík), 2 ♂; Toparlar, pasture, YPWT, 37°03'09"N, 28°20'17"E, 4 m, 13.–14.ix.2014 (Barták & Kubík), 9 ♂; **SA**: Samsun University campus, 41°22'N, 36°11'E, 22vi.–4.vii.2014 (Barták & Kubík), 14 ♂ (CULSP).

**DI**: European-Anatolian.

**DIT**: MG**, SA**.

**Helicophagella (s. str.) noverca (Rondani, 1860)**

**Material examined. SA**: Samsun University campus, 41°22'N, 36°11'E, 22vi.–4.vii.2014 (Barták & Kubík), 5 ♂ (CULSP).

**DI**: Mediterranean.

**DIT**: **ES** (Aslan and Çalışkan 2009), SA**.

**Helicophagella (s. str.) novercoides (Böttcher, 1913)**

**Material examined. MG**: Muğla University campus, SW+PT, 700 m, 37°09'41"N, 28°22'21"E, 29.iv.–10.v.2013 (Barták & Kubík), 2 ♂; Akyaka, salty meadow, 2 m, PT, 37°01'52"N, 28°20'00"E, 27.iv.–1.v.2016 (Barták & Kubík), 1 ♂; Muğla – 13 km NE, pine wood + pasture, 1100–1300 m, 37°15'N, 28°30'E, 2–3.v.2016 (Barták & Kubík), 1 ♂ (CULSP).

**DI**: West Palaearctic.

**DIT**: **TR** (Koçak 2014; Koçak and Kemal 2012): **ANT** (Kara and Pape 2002; Koçak and Kemal 2009, 2013, 2015), **ERZ** (Pekbey 2011), MG**.

**Helicophagella (Parabellieria) dreyfusi (Lehrer, 1994), comb. n.**

*Ahavanella dreyfusi* Lehrer, 1994

**DI**: West Palaearctic-Oriental.

**DIT**: **TR** (Kara and Pape 2002; Koçak 2014; Koçak and Kemal 2012, 2013, 2015).

##### Subtribe Phytosarcophagina

**Phytosarcophaga (s. str.) destructor (Malloch, 1929)**

**Material examined. AY**: 8 km S of Çine, river bank, 68 m, 37°32'34"N, 28°03'46"E, 28.–30.vi.2015 (Barták & Kubík), 1 ♂; ibid., 29.iv.–1.v.2016 (Barták & Kubík), 1 ♂ (CULSP).

**DI**: West Palaearctic-Afrotropical.

**DIT**: **TR** (Koçak 2014; Koçak and Kemal 2012), **AD** (Kara and Pape 2002), AY**, **MN** (Kara and Pape 2002; Koçak and Kemal 2009, 2013, 2015), **ME** (Kara and Pape 2002; Koçak and Kemal 2009, 2013, 2015).

##### Subtribe Heteronychiina

**Heteronychia (Boettcherella) helenae (Trofimov, 1948)**

**Material examined. MG**: Akyaka, pasture, YPWT, 37°03'09"N, 28°20'17"E, 4 m, 13.–14.ix.2014 (Barták & Kubík), 5 ♂ (CULSP).

**DI**: Central Palaearctic.

**DIT**: **TR** (Koçak 2014; Koçak and Kemal 2012): **ANT** (Verves and Khrokalo 2015), **ERZ** (Pekbey 2011; Pekbey and Hayat 2011, 2013), **IZ** (Koçak and Kemal 2015; Whitmore 2011), MG**.

**Heteronychia (Boettcherella) setinervis (Rondani, 1860)**

**Material examined. MG**: Akyaka, salty meadow, 2 m, 37°01'49"N, 28°20'01"E, 22.vi.–1.vii.2015 (Barták & Kubík), 4 ♂ (CULSP).

**DI**: West-Central Palaearctic.

**DIT**: **TR** (Kara and Pape 2002; Koçak 2014; Koçak and Kemal 2012): **ANT** (Verves and Khrokalo 2015), **DE** (Koçak and Kemal 2015; Lehrer 1977), **ERZ** (Pekbey 2011; Pekbey and Hayat 2011), **GA** (Whitmore 2010), **HT** (Koçak and Kemal 2015; Whitmore 2010), **KY** (Hayat et al. 2008; Koçak and Kemal 2009, 2015), **KN** (Verves and Khrokalo 2015), **ME** (Koçak and Kemal, 2015; Verves and Khrokalo 2015; Whitmore 2010), MG**, **SN** (Koçak and Kemal 2015), **TO** (Koçak and Kemal 2015; Whitmore 2010).

**Heteronychia (Ctenodasypygia) minima (Rondani, 1862)**

*Leclercqiomyia mousseti* Lehrer, 1976a: 200.

**Material examined. MG**: Akyaka, pasture, YPWT, 37°03'09"N, 28°20'17"E, 4 m, 13.–14.ix.2014 (Barták & Kubík), 14 ♂; ibid., salty meadow, SW+PT, 37°02'53"N, 28°19'39"E, 28.iv.–9.v.2013 (Barták & Kubík), 9 ♂; ibid., pasture, 4 m, 37°03'09"N, 28°20'17"E, 8.–14.ix.2014 (Barták & Kubík), 10 ♂; ibid., salty meadow, 2 m, 37°01'49"N, 28°20'01"E, 22.vi.–1.vii.2015 (Barták & Kubík), 19 ♂; ibid., PT, 37°01'62"N, 28°20'00"E, 27.iv.–1.v.2016 (Barták & Kubík), 3 ♂; Dalyan, farm, 1 m, MT, 36°48'54"N, 28°39'04"E, 8.–20.viii.2015 (Dursun), 3 ♂; ibid., salty meadow, PT, 36°47'49"N, 28°38'55"E, 28.–30.iv.2016 (Barták & Kubík), 4 ♂; Muğla University campus, MT, 730 m, 37º09’38"N, 28º22’11"E, 19.xiii.-17.ix.2015 (H. Kavak), 4 ♂ (CULSP).

**DI**: European-Western Midle East.

**DIT**: **TR** (Koçak 2014; Koçak and Kemal 2012), **AY** (Koçak and Kemal 2013, 2015), **GA** (Lehrer 1976a), **IZ** (Koçak and Kemal 2013, 2015), MG**.

**Heteronychia (Ctenodasypygia) siciliensis (Böttcher, 1913)**

**Material examined. SN**: Birecik, E from Gaziantep, pastures SE from town, 37.00N/38.00E, 24–25.iv.1997 (Vrabec V.), ♂ (CULSP).

**DI**: Mediterranean.

**DIT**: **TR** (Kara and Pape 2002; Koçak 2014; Koçak and Kemal 2009, 2012): **ANT** (Verves & Khrokalo 2015), **AY** (Lehrer 1976a), **IZ** (Koçak & Kemal 2015, Whitmore 2011), SN**.

**Heteronychia (Pandelleola) boettcheri (Villeneuve, 1911)**

*Pandelleola taurica*: Lehrer 2008: 1.

**Material examined. AY**: 8 km S of Çine river bank, 68 m, 37°32'34"N, 28°03'46"E, 10.–12.ix.2014 (Barták & Kubík), 3 ♂; ibid., 28.–30.vi.2015 (Barták & Kubík), 10 ♂; ibid., SW, 29.iv.–1.v.2016 (Barták & Kubík), 3 ♂, **MG**: Akyaka, pasture, YPWT, 37°03'09"N, 28°20'17"E, 4 m, 13.–14.ix.2014 (Barták & Kubík), 1 ♂; ibid., 8.–14.ix.2014 (Barták & Kubík), 3 ♂ (CULSP).

**DI**: European-Mid Eastern.

**DIT**: **TR** (Kara and Pape 2002 – as Sarcophaga (Heteronychia) taurica; Koçak 2014; Koçak and Kemal 2009 – as Sarcophaga (Heteronychia) taurica, 2012): **AM** (Koçak and Kemal 2013, 2015; Whitmore 2011), **ANT** (Verves & Khrokalo 2015), AY**, **BO** (Lehrer 1977 – as Heteronychia (Pandelleola) gaspari), **DU** (Koçak & Kemal 2015), **ER** (Pekbey 2011; Pekbey and Hayat 2011, 2013°), **ERZ** (Pekbey 2011; Pekbey and Hayat 2011, 2013°), **ME** (Koçak and Kemal 2013, 2015; Whitmore 2011), MG**, **SA** (Koçak and Kemal 2013, 2015; Whitmore 2011), **TO** (Koçak and Kemal 2013, 2015; Whitmore 2011).

**Heteronychia (Pandelleola) filia (Rondani, 1860)**

**Material examined. AY**: 8 km S of Çine, river bank, 68 m, 37°32'34"N, 28°03'46"E, 10.–12.ix.2014 (Barták & Kubík), 1 ♂; ibid., 28.–30.vi.2015 (Barták & Kubík), 6 ♂; ibid., 29.iv.–1.v.2016 (Barták & Kubík), 3 ♂; **MG**: Akyaka, pasture, YPWT, 37°03'09"N, 28°20'17"E, 4 m, 13.–14.ix.2014 (Barták & Kubík), 6 ♂; ibid., 8.–14.ix.2014 (Barták & Kubík), 1 ♂; ibid., salty meadow, 2 m, PT, 37°01'49"N, 28°20'01"E, 22.vi.–1.vii.2015 (Barták & Kubík), 1 ♂; 4 km N of Yatağan, flowers, 460 m, 37°22'12"N, 28°09'22"E, 30.vi.2016 (Barták & Kubík), 1 ♂; Kızılyaka, on flowers, 105 m, 37°01'21"N, 28°26'18"E, 27.iv.–4.v.2016 (Barták & Kubík), 1 ♂; **SA**: Samsun University campus, 41°22'N, 36°11'E, 22vi.–4.vii.2014 (Barták & Kubík), 1 ♂ (CULSP).

**DI**: West Palaearctic.

**DIT**: **TR** (Koçak 2014; Koçak and Kemal 2012), **AM** (Kara and Pape 2002; Koçak and Kemal 2009, 2013, 2015; Whitmore 2011), **ANT** (Whitmore 2011; Kara and Pape 2002; Koçak and Kemal 2009, 2013, 2015; Verves and Khrokalo 2015; Whitmore 2011), AY**, **BY** (Pekbey 2011; Pekbey and Hayat 2011), **ER** (Pekbey 2011; Pekbey and Hayat 2011), **ERZ** (Pekbey 2011; Pekbey and Hayat 2011), **ES** (Aslan 2006; Aslan and Çalışkan 2009; Koçak and Kemal 2009, 2013, 2015), **KY** (Hayat et al. 2008; Koçak and Kemal 2009, 2015), MG**, **SA** (Kara and Pape 2002; Koçak and Kemal 2009, 2015), **TO** (Kara and Pape 2002; Koçak and Kemal 2009, 2013, 2015; Whitmore 2011), **TB** (Hayat et al. 2008; Koçak and Kemal 2009, 2013, 2015).

**Heteronychia (s. str.) bulgarica (Enderlein, 1936)**

**Material examined. SA**: Samsun University campus, 41°22'N, 36°11'E, 22vi.–4.vii.2014 (Barták & Kubík), 1 ♂ (CULSP).

**DI**: European-Mid Eastern.

**DIT**: **BY** (Pekbey 2011; Pekbey and Hayat 2011, 2013a), **ER** (Pekbey and Hayat 2011, 2013a), **ERZ** (Pekbey 2011; Pekbey and Hayat 2011, 2013a), SA**.

**Heteronychia (s. str.) haemorrhoides (Böttcher, 1913)**

*Heteronychia wahisi* Lehrer, 1976b: 264.

**Material examined. AY**: 8 km S of Çine, river bank, SW, 68 m, 37°32'34"N, 28°03'46"E, 28.–30.vi.2015 (Barták & Kubík), 1 ♂; **MG**: Akyaka, pasture, 6 m, 37°03'19"N, 28°20'07"E, YPWT, 28.iv.–8.v.2013, (Barták & Kubík), 4 ♂; ibid., forest, 30 m, YPWT, 37°03'16"N, 28°19'35"E, 30.iv.–9.v.2013, (Barták & Kubík), 1 ♂; ibid., pasture, YPWT, 37°03'09"N, 28°20'17"E, 4 m, 13.–14.ix.2014 (Barták & Kubík), 5 ♂; **SA**: Samsun University campus, 41°22'N, 36°11'E, 22vi.–4.vii.2014 (Barták & Kubík), 2 ♂ (CULSP).

**DI**: European-Middle East-Mid Asiatic.

**DIT**: **TR** (Kara and Pape 2002; Koçak 2014; Koçak and Kemal 2009, 2012), **AM** (Koçak and Kemal 2013, 2015; Whitmore 2010), AY**, **ER** (Pekbey 2011; Pekbey and Hayat 2011, 2013°), **ERZ** (Pekbey 2011; Pekbey and Hayat 2011, 2013°), **HT** (Koçak and Kemal 2013, 2015; Lehrer 1976b), MG**, SA**, **TO** (Koçak and Kemal 2013, 2015; Whitmore 2010).

Heteronychia (s. str.) infixa (Böttcher, 1913)*

**Material examined. SA**: Samsun University campus, 41°22'N, 36°11'E, 22vi.–4.vii.2014 (Barták & Kubík), 1 ♂ (CULSP).

**DI**: European-Anatolian.

**DIT**: SA**.

**Heteronychia (s. str.) kerteszi (Villeneuve, 1912)**

**Material examined. MG**: Muğla University campus, 700 m, SW+PT, 37°09'41"N, 28°22'21"E, 29.iv.–10.v.2013 (Barták & Kubík), 3 ♂; ibid., 700 m, 37°09'42"N, 28°22'22"E (O.Dursun), iv–v.2014, 1 ♂; ibid., 720 m, MT, 37°09'42"N, 28°22'13"E, iv.2015 (H. Kavak), 1 ♂; ibid., 26–27.vi.2015 (Barták & Kubík), 2 ♂; Muğla – 13 km NE, pine wood, 1200 m, 37°14'50"N, 28°30'00"E, 23–27.v.i.2015 (Barták & Kubík), 1 ♂ (CULSP).

**DI**: East Mediterranean.

**DIT**: **TR** (Koçak 2014; Koçak and Kemal 2012), **ANT** (Kara and Pape 2002; Koçak and Kemal 2009, 2013, 2015), **IZ** (Koçak and Kemal 2013, 2015; Whitmore 2011), MG**.

**Heteronychia (s. str.) lacrymans (Villeneuve, 1912)**

**Material examined. MG**: Akyaka, pasture, YPWT, 37°03'09"N, 28°20'17"E, 4 m, 13.–14.ix.2014 (Barták & Kubík), 6 ♂ (CULSP).

**DI**: East Mediterranean.

**DIT**: **TR** (Koçak 2014; Koçak and Kemal 2012), **AF** (Kara and Pape 2002 – as Sarcophaga (Heteronychia) zhelochovtzevi; Koçak and Kemal 2009 – as Sarcophaga (Heteronychia) zhelochovtzevi, 2013, 2015; Whitmore 2011), **ER** (Pekbey 2011; Pekbey and Hayat 2011, 2013°), **ERZ** (Pekbey 2011; Pekbey and Hayat 2011, 2013°), MG**.

Heteronychia (s. str.) pontica (Rohdendorf, 1937)*

**Material examined. SA**: Samsun University campus, 41°22'N, 36°11'E, 22vi.–4.vii.2014 (Barták & Kubík), 13 ♂ (CULSP).

**DI**: East Mediterranean.

**DIT**: SA**.

Heteronychia (s. str.) porrecta (Böttcher, 1913)*

**Material examined. SA**: Samsun University campus, 41°22'N, 36°11'E, 22vi.–4.vii.2014 (Barták & Kubík), 1 ♂ (CULSP).

**DI**: European-Anatolian.

**DIT**: SA**.

**Heteronychia (s. str.) schineri (Bezzi, 1891)**

**Material examined. SA**: Samsun University campus, 41°22'N, 36°11'E, 22vi.–4.vii.2014 (Barták & Kubík), 1 ♂.

**DI**: European-Mid Eastern.

**DIT**: **TR** (Koçak 2014), **AM** (Kara and Pape 2002; Koçak and Kemal 2009, 2012, 2013, 2015), **BY** (Pekbey 2011; Pekbey and Hayat 2011, 2013°), SA**, **TO** (Kara and Pape 2002; Koçak and Kemal 2009, 2013, 2015).

*Karovia hirticrus* (Pandellé, 1896)*

**Material examined. SA**: Samsun University campus, 41°22'N, 36°11'E, 22.vi.–4.vii.2014 (Barták & Kubík), 1 ♂ (CULSP).

**DI**: West Palaearctic.

**DIT**: SA**.

##### Subtribe Phallanthina

*Bellieriomima subulata* (Pandellé, 1896)*

**Material examined. SA**: Samsun University campus, 41°22'N, 36°11'E, 22vi.–4.vii.2014 (Barták & Kubík), 4 ♂ (CULSP).

**DI**: European-Siberian-Central Asiatic.

**DIT**: SA**.

Myorhina (s. str.) lunigera (Böttcher, 1914)*

**Material examined. SA**: Samsun University campus, 41°22'N, 36°11'E, 22vi.–4.vii.2014 (Barták & Kubík), 1 ♂ (CULSP).

**DI**: European-Mid Eastern.

**DIT**: SA**.

**Myorhina (s. str.) nigriventris (Meigen, 1826)**

**Material examined. MG**: Akyaka, forest, 30 m, 37°03'16"N, 28°19'35"E, 30.iv.–9.v.2013, YPWT (Barták & Kubík), 1 ♂; ibid., pasture, 8 m, 37°03'11"N, 28°20'33"E, 27.iv.2016 (Barták & Kubík), 1 ♂; 4 km N of Yatağan, flowers, 460 m, 37°22'12"N, 28°09'22"E, 30.vi.2016 (Barták & Kubík), 2 ♂; **AY**: 8 km S of Çine, river bank, SW, 68 m, 37°32'34"N, 28°03'46"E, 28.–30.vi.2015 (Barták & Kubík), 1 ♂; ibid., 28.–30.vi.2015 (Barták & Kubík), 3 ♂; ibid., PT, 37°32'34"N, 28°03'46"E, 29.iv.–1.v.2016 (Barták & Kubík), 1 ♂; **SA**: Samsun University campus, 41°22'N, 36°11'E, 22vi.–4.vii.2014 (Barták & Kubík), 8 ♂ (CULSP).

**DI**: West Palaearctic.

**DIT**: **TR** (Koçak 2014; Koçak and Kemal 2012): **AM** (Kara and Pape 2002; Koçak and Kemal 2009, 2013, 2015), **BY** (Pekbey 2011), **ER** (Pekbey 2011), **ERZ** (Pekbey 2011; Pekbey and Hayat 2010), MG**, SA**, **TO** (Kara and Pape 2002; Koçak and Kemal 2009, 2013, 2015).

Myorhina (s. str.) socrus (Rondani, 1860)*

**Material examined. MG**: Muğla – 13 km NE, pine wood, 1200 m, 37°14'50"N, 28°30'00"E, 23–27.v.i.2016 (Barták & Kubík), 2 ♂, 1 ♀; Toparlar, 8 m, lowland forest, SW+PT, 36°58'39"N, 28°39'30"E, 28.–30.iv.2016 (Barták & Kubík), 1 ♂ (CULSP).

**DI**: European-Mid Eastern.

**DIT**: MG**.

**Myorhina (s. str.) soror (Rondani, 1860)**

**Material examined. AY**: 8 km S of Çine, river bank, 68 m, PT, 37°32'34"N, 28°03'46"E, 29.iv.–1.v.2016 (Barták & Kubík), 1 ♂; **MG**: Akyaka, pasture, 6 m, 37°03'19"N, 28°20'07"E, YPWT, 28.iv.–8.v.2013, (Barták & Kubík), 2 ♂; ibid., YPWT, 37°03'09"N, 28°20'17"E, 4 m, 13.–14.ix.2014 (Barták & Kubík), 10 ♂; ibid., 37°03'09"N, 28°20'17"E, 8.–14.ix.2014 (Barták & Kubík), 2 ♂; **SA**: Samsun University campus, 41°22'N, 36°11'E, 22vi.–4.vii.2014 (Barták & Kubík), 13 ♂ (CULSP).

**DI**: Westpalaearctic.

**DIT**: **TR** (Koçak 2014; Koçak and Kemal 2012): **AM** (Kara and Pape 2002; Koçak and Kemal 2009, 2013, 2015), AY**, **BY** (Pekbey 2011), **ER** (Pekbey 2011), **ERZ** (Pekbey 2011), MG**, **SA** (Kara and Pape 2002; Koçak and Kemal 2009, 2013, 2015).

***Pandelleana protuberans* (Pandellé, 1896)**

**Material examined. ANT**: Güzelsu nr Akseki, 5.vi.2005 (C. Bystrowski), 1 ♂ (CULSP).

**DI**: European-Siberian-Centralasiatic.

**DIT**: **TR** (Koçak 2014; Koçak and Kemal 2012, 2013, 2015, “Anatolia”: Rohdendorf 1937), ANT**, **ERZ** (Pekbey 2011), **ES** (Aslan and Çalışkan 2009).

***Pandelleana tahtaliana* Lehrer, 2004**

**Material examined. MG**: Muğla University campus, SW+PT, 700 m, 37°09'41"N, 28°22'21"E, 29.iv.–10.v.2013 (Barták & Kubík), 1 ♂; Muğla – 13 km NE, pine wood + pasture, 1100–1300 m, 37°15'N, 28°30'E, 2–3.v.2016 (Barták & Kubík), 1 ♂ (CULSP).

**DI**: Anatolian.

**DIT**: **KY** (Lehrer 2004), **KN** (Lehrer 2004), MG**.

***Pseudothyrsocnema spinosa* (Villeneuve, 1912)**

**Material examined. AY**: 8 km S of Çine river bank, 68 m, 37°32'34"N, 28°03'46"E, 10.–12.ix.2014 (Barták & Kubík), 1 ♂; ibid., 28.–30.vi.2015 (Barták & Kubík), 3 ♂; ibid., PT, 37°32'34"N, 28°03'46"E, 29.iv.–1.v.2016 (Barták & Kubík), 1 ♂; **MG**: Akyaka, pasture, 6 m, 37°03'19"N, 28°20'07"E, 28.iv.–8.v.2013, YPWT (Barták & Kubík), 4 ♂; ibid., salty meadow, SW+PT, 37°02'53"N, 28°19'39"E, 28.iv.–9.v.2013 (Barták & Kubík), 1 ♂; ibid., pasture, YPWT, 37°03'09"N, 28°20'17"E, 4 m, 13.–14.ix.2014 (Barták & Kubík), 4 ♂; ibid., pasture, 4 m, 37°03'09"N, 28°20'17"E, 8.–14.ix.2014 (Barták & Kubík), 2 ♂; ibid., salty meadow, 2 m, PT, 37°01'62"N, 28°20'00"E, 27.iv.–1.v.2016 (Barták & Kubík), 1 ♂; Dalyan, farm, MT, 1 m, 36°48'54"N, 28°39'04"E, 8.–20.viii.2015 (Dursun), 1 ♀; ibid., salty meadow, PT, 36°47'49"N, 28°38'55"E, 28.–30.iv.2016 (Barták & Kubík), 3 ♂ (CULSP).

**DI**: European-Mid Eastern.

**DIT**: **TR** (Koçak 2014; Koçak and Kemal 2012), **AD** (Kara and Pape 2002; Koçak and Kemal 2009, 2013, 2015), AY**, MG**.

***Sarina sexpunctata* (Fabricius, 1805)**

**Material examined. SA**: Samsun University campus, 41°22'N, 36°11'E, 22vi.–4.vii.2014 (Barták & Kubík), 1 ♂ (CULSP).

**DI**: Palaearctic.

**DIT**: **TR** (Koçak 2014; Koçak and Kemal 2012), **BY** (Pekbey 2011), **ERZ** (Pekbey 2011), SA**, **TO** (Kara and Pape 2002; Koçak and Kemal 2009, 2013, 2015).

***Thyrsocnema incisilobata* (Pandellé, 1896)**

**Material examined.** MG: Muğla – 13 km NE, pine wood, 1200 m, 37°14'50"N, 28°30'00"E, 23–27.v.i.2016 (Barták & Kubík), 1 ♂; Toparlar, 8 m, lowland wood, 36°58'27"N, 28°38'50"E, 22.–24.vi.2015 (Barták & Kubík), 1 ♂; SA: Samsun University campus, 41°22'N, 36°11'E, 22vi.–4.vii.2014 (Barták & Kubík), 3 ♂ (CULSP).

**DI**: Palaearctic.

**DIT**: **TR** (Koçak 2014; Koçak and Kemal 2012), **AM** (Kara and Pape 2002; Koçak and Kemal 2009, 2013, 2015), **ERZ** (Pekbey 2011), MG**, SA**, **TO** (Kara and Pape 2002; Koçak and Kemal, 2009, 2013, 2015).

##### Subtribe Parasarcophagina

***Bercaea africa* (Wiedemann, 1824)**

**Material examined. MG**: Akyaka, pasture, 6 m, 37°03'19"N, 28°20'07"E, 28.iv.–8.v.2013, YPWT (Barták & Kubík), 1 ♂; ibid., forest, 30 m, 37º03’16˝ N, 28º19’35˝ E, 30.iv.-9.v.2013, YPWT (Barták & Kubík), 1 ♂; Muğla, protein trap, pine wood, 700 m, 37°09'41"N, 28°22'21"E, xi.–iii.2013 (Barták & Kubík), 2 ♂ (CULSP).

**DI**: Cosmopolitan.

**DIT**: **TR** (Kara and Pape 2002; Koçak 2014; Koçak and Kemal 2012 – both Sarcophaga (Bercaea) africa & Sarcophaga (Bercaea) cruentata), **BT** (Koçak and Kemal 2013, 2015 – both Sarcophaga (Bercaea) africa & Sarcophaga (Bercaea) cruentata), **BY** (Pekbey 2011), **DB** (İpek et al. 2009, 2011 – as *Sarcophaga haemorrhoidalis*), **ED** (Çoban and Beyarslan 2013), **EL** (Şaki and Özer 1999a, b – as *Sarcophaga haemorrhoidalis*), **ERZ** (Pekbey 2011; Pekbey and Hayat 2010), **ES** (Aslan 2006; Aslan and Çalışkan 2009; Koçak and Kemal 2009, 2013, 2015 – both Sarcophaga (Bercaea) africa & Sarcophaga (Bercaea) cruentata), **KAR** (Hayat et al. 2008; Koçak and Kemal 2009, 2013, 2015 – both Sarcophaga (Bercaea) africa & Sarcophaga (Bercaea) cruentata), **KI** (Dik et al. 2012 – as *Sarcophaga haemorrhoidalis*), **KN** (Dik et al. 2012 – as *Sarcophaga haemorrhoidalis*), **ME** (Aslan 2006; Kara and Pape 2002), MG**, **SN** (Sevgili et al. 2004), **TO** (Aslan 2006; Kara and Pape 2002), **VA** (Koçak and Kemal 2015 2015 – both Sarcophaga (Bercaea) africa & Sarcophaga (Bercaea) cruentata; Özdal and Değer 2005 – as *Sarcophaga haemorrhoidalis*).

***Liopygia*** (***Engelisca***) *surcoufi* (Villeneuve, 1913)*

**Material examined. SA**: Samsun University campus, 41°22'N, 36°11'E, 22vi.–4.vii.2014 (Barták & Kubík), 1 ♂ (CULSP).

**DI**: Mediterranean.

**DIT**: SA**.

***Liosarcophaga*** (***Curranea***) ***tibialis* (Macquart, 1851)**

**Material examined. AY**: 8 km S of Çine river bank, 68 m, 37°32'34"N, 28°03'06"E, 10.–12.ix.2014 (Barták & Kubík), 1 ♂; **MG**: Akyaka, pasture, 6 m, 37°03'19"N, 28°20'07"E, 28.iv.–8.v.2013, YPWT (Barták & Kubík), 3 ♂; ibid., YPWT, 37°03'09"N, 28°20'17"E, 4 m, 13.–14.ix.2014 (Barták & Kubík), 3 ♂; ibid., salty meadow, 2 m, 37°01'49"N, 28°20'01"E, 22.vi.–1.vii.2015 (Barták & Kubík), 1 ♂; Muğla University campus, 720 m, MT, 37°09'42"N, 28°22'13"E, 26–27.vi.2015 (Barták & Kubík), 1 ♂; ibid., protein trap, pine wood, 700 m, 37°09'41"N, 28°22'21"E, xi.–iii.2013 (Barták & Kubík), 2 ♂; Dalyan, salty meadow, PT, 36°47'49"N, 28°38'55"E, 28.–30.iv.2016 (Dursun), 1 ♂; **SA**: Samsun University campus, 41°22'N, 36°11'E, 22vi.–4.vii.2014 (Barták & Kubík), 1 ♂ (CULSP).

**DI**: West Palaearctic-Afrotropical-Oriental-Australasian/Oceanian.

**DIT**: **AN** (Açikgöz et al., 2011), AY**, MG**, SA**, **SN** (Sevgili et al. 2004).

**Liosarcophaga (Pandelleisca) similis (Meade, 1876)**

**Material examined. AY**: 8 km S of Çine river bank, 68 m, 37°32'34"N, 28°03'46"E, 10.–12.ix.2014 (Barták & Kubík), 1 ♂; **MG**: Akyaka, pasture, 6 m, 37°03'19"N, 28°20'07"E, 28.iv.–8.v.2013, YPWT (Barták & Kubík), 1 ♂; Toparlar, lowland wood, 8 m, 36°59'27"N, 28°38'50"E, 22.–24.vi.2015 (Barták & Kubík), 1 ♂ (CULSP).

**DI**: Transpalaearctic-Oriental.

**DIT**: **TR** (Koçak 2014; Koçak and Kemal 2012), AY**, MG**, **TB** (Kara and Pape 2002; Koçak and Kemal 2009, 2013, 2015).

**Liosarcophaga (s. str.) emdeni (Rohdendorf, 1969)**

**Material examined. SA**: Samsun University campus, 41°22'N, 36°11'E, 22vi.–4.vii.2014 (Barták & Kubík), 9 ♂ (CULSP).

**DI**: European-Siberian-Central Asiatic.

**DIT**: **TR** (Koçak 2014; Koçak and Kemal 2012), **AM** (Kara and Pape 2002; Koçak and Kemal 2009, 2013, 2015), **ER** (Pekbey 2011), **ERZ** (Pekbey 2011), SA**.

**Liosarcophaga (s. str.) jacobsoni (Rohdednorf, 1937)**

**Material examined. MG**: Akyaka, pasture, YPWT, 37°03'19"N, 28°20'07"E, 6 m, 28.iv.–8.v.2013 (Barták & Kubík), 1 ♂; ibid., salty meadow, SW+PT, 37°02'53"N, 28°19'39"E, 28.iv.–9.v.2013 (Barták & Kubík), 2 ♂; ibid., pasture, YPWT, 37°03'09"N, 28°20'17"E, 4 m, 13.–14.ix.2014 (Barták & Kubík), 1 ♂; ibid., salty meadow, 2 m, 37°01'49"N, 28°20'01"E, 22.vi.–1.vii.2015 (Barták & Kubík), 4 ♂; ibid., PT, 37°01'52"N, 28°20'00"E, 27.iv.–1.v.2016 (Barták & Kubík), 2 ♂ (CULSP).

**DI**: Transpalaearctic subboreal.

**DIT**: **ERZ** (Pekbey 2011; Pekbey and Hayat 2010), **ES** (Aslan, 2006; Aslan and Çalışkan 2009), MG**.

**Liosarcophaga (s. str.) portschinskyi (Rohdendorf, 1937)**

**Material examined. SA**: Samsun University campus, 41°22'N, 36°11'E, 22vi.–4.vii.2014 (Barták & Kubík), 3 ♂ (CULSP).

Distribution: Transpalaearctic-Oriental.

**DIT**: **TR** (Kara and Pape 2002; Koçak 2014; Koçak and Kemal 2009, 2012, 2015), **ERZ** (Pekbey 2011), **ES** (Aslan and Çalışkan 2009), SA**.

**Parasarcophaga (s. str.) albiceps (Meigen, 1826)**

**Material examined. SA**: Samsun University campus, 41°22'N, 36°11'E, 22vi.–4.vii.2014 (Barták & Kubík), 1 ♂ (CULSP).

**DI**: Transpalaearctic- Oriental-Australasian/Oceanian.

**DIT**: **TR** (Kara and Pape 2002; Koçak 2014; Koçak and Kemal 2009, 2012, 2015), **ERZ** (Pekbey 2011), SA**.

##### Subtribe Boettcheriscina

***Rosellea aratrix* (Pandellé, 1896)**

**Material examined. SA**: Samsun University campus, 41°22'N, 36°11'E, 22vi.–4.vii.2014 (Barták & Kubík), 2 ♂ (CULSP).

**DI**: Transpalaearctic-Nearctic-Oriental.

**DIT**: **TR** (Koçak 2014; Koçak and Kemal 2012), **BU** (Kara and Pape 2002; Koçak and Kemal 2009, 2013, 2015), SA**.

*Rosellea beckiana* Lehrer, 1996*

**Material examined. AY**: 8 km S of Çine, river bank, SW, 68 m, 37°32'34"N, 28°03'46"E, 28.–30.vi.2015 (Barták & Kubík), 1 ♂; ibid., 29.iv.–1.v.2016 (Barták & Kubík), 1 ♂; **MG**: Akyaka, pasture, YPWT, 37°03'09"N, 28°20'17"E, 4 m, 13.–14.ix.2014 (Barták & Kubík), 2 ♂; Muğla, protein trap, pine wood, 700 m, 37°09"41"N, 28°22"21"E, xi.–iii.2013 (Barták & Kubík), 1 ♂; Toparlar, lowland wood, 8 m, 36°59'27"N, 28°38'50"E, 22.–24.vi.2015 (Barták & Kubík), 2 ♂; ibid., SW+PT, 36°58'39"N, 28°39'30"E, 28.–30.iv.2016 (Barták & Kubík), 1 ♂ (CULSP).

**DI**: East Mediterranean.

**DIT**: AY**, MG**.

##### Subtribe Sarcophagina

***Sarcophaga lehmanni* Müller, 1922**

**Material examined. AY**: 8 km S of Çine, river bank, SW, 68 m, 37°32'34"N, 28°03'46"E, 28.–30.vi.2015 (Barták & Kubík), 2 ♂; **HA**: 25 km E of Ġözeldere, 37°32'N, 43°49'E, 930 m, 22.vi.2010 (Mi. Halada), 1 ♂; **MG**: Akyaka, forest, 30 m, 37°03'16"N, 28°19'35"E, 30.iv.–9.v.2013, YPWT (Barták & Kubík), 1 ♂; ibid., pasture, 4 m, 37°03'09"N, 28°20'17"E, 8.–14.ix.2014 (Barták & Kubík), 2 ♂; Muğla University campus, SW+PT, 700 m, 37°09'41"N, 28°22'21"E, 29.iv.–10.v.2013 (Barták & Kubík), 1 ♂; ibid., 37°09'42"N, 28°22'22"E (O.Dursun), iv.–v.2014, 1 ♂; ibid., MT, 730 m, 37°09'38"N, 28°22'11"E, 19.viii.–17.ix.2015 (H. Kavak), 1 ♂; Dalyan, farm, MT, 1 m, 36°48'54"N, 28°39'04"E, 8.–20.viii.2015 (Dursun), 1 ♂; ibid., salty meadow, PT, 36°47'49"N, 28°38'55"E, 28.–30.iv.2016 (Barták & Kubík), 1 ♂; **SA**: Samsun University campus, 41°22'N, 36°11'E, 22vi.–4.vii.2014 (Barták & Kubík), 3 ♂ (CULSP).

**DI**: West-Central Palaearctic.

**DIT**: **TR** (Kara and Pape 2002; Koçak 2014; Koçak and Kemal 2012 – as Sarcophaga (s. str.) lasiostyla), **AM** (Aslan 2006), AY**, **BY** (Pekbey 2011), **ER** (Pekbey 2011), **ERZ** (Pekbey 2011; Pekbey and Hayat 2010), **ES** (Aslan, 2006; Aslan and Çalışkan 2009; Koçak and Kemal 2009 – as Sarcophaga (s. str.) lasiostyla, 2013, 2015 – as Sarcophaga (s. str.) lasiostyla), HA**, **IG** (Hayat et al. 2008; Koçak and Kemal 2009 – as Sarcophaga (s. str.) lasiostyla, 2013, 2015 – as Sarcophaga (s. str.) lasiostyla), **IZ** (Civelek and Tezcan 2005; Koçak and Kemal 2009 – as Sarcophaga (s. str.) lasiostyla, 2013, 2015 – as Sarcophaga (s. str.) lasiostyla), **KAR** (Hayat et al. 2008; Koçak and Kemal 2009 – as Sarcophaga (s. str.) lasiostyla, 2013, 2015 – as Sarcophaga (s. str.) lasiostyla), **KY** (Hayat et al. 2008; Koçak and Kemal 2009 – as Sarcophaga (s. str.) lasiostyla, 2013, 2015 – as Sarcophaga (s. str.) lasiostyla), **MN** (Civelek and Tezcan 2005; Koçak and Kemal 2009 – as Sarcophaga (s. str.) lasiostyla, 2013, 2015 – as Sarcophaga (s. str.) lasiostyla), MG**, SA**.

## Discussion

This paper presents the results of an intensive collecting effort by two of the authors (MB and SK) in Turkey between 2011 and 2016. It adds new faunistic records for 68 species. The following 22 species newly recorded for Turkey are presented: *Apodacra dispar*, *Bellieriomima subulata*, *Blaesoxipha confusa*, *Craticulina barbifera*, *Helicophagella macrura*, *H. novella*, *Heteronychia infixa*, *H. pontica*, *H. porrecta*, *Karovia hirticrus*, *Liopygia surcoufi*, *Metopia grandii*, *Miltogramma aurifrons*, *M. brevipila*, *M. testaceifrons*, *M. turkmenora*, *Myorhina lunigera*, *M. socrus*, *Paragusia multipunctata*, *Rosellea beckiana*, *Sarcophila canaanita*, and *Sphecapatodes ornatus*. A further 46 species are recorded for the first time from at least one Turkish province.

Previously, 132 species of Sarcophagidae were listed from Turkey (Hayat et al. 2008; Kara and Pape 2002; Koçak 2014; Koçak and Kemal 2009, 2012, 2013, 2015; Pape et al. 2015; Pekbey 2011; Pekbey and Hayat 2011; Whitmore, 2011; Whitmore et al. 2013). Our findings increase this number to 154. This relatively large number of faunistic novelties indicates that there is a low degree of faunistic research on this family in Turkey.
